# Erosive Wear Mitigation Using 3D-Printed Twisted Tape Insert Under Liquid–Solid Flow

**DOI:** 10.3390/ma19030453

**Published:** 2026-01-23

**Authors:** Hammad Subhani, Rehan Khan, Darko Damjanović

**Affiliations:** 1 Department of Mechanical Engineering, College of Electrical and Mechanical Engineering, National University of Sciences and Technology, Islamabad 44000, Pakistan; hammadsubhani1320@gmail.com; 2 Mechanical Engineering Faculty in Slavonski Brod, University of Slavonski Brod, Ulica 108. brigade ZNG 36, 35 000 Slavonski Brod, Croatia; ddamjanovic@unisb.hr

**Keywords:** erosion mitigation, twisted tape optimization, additive manufacturing, slurry transport, abrasive flow control, pipe durability

## Abstract

This study examines whether twisted tape inserts in a pipe system can reduce pipe erosion under a liquid–solid flow regime. Three different twisted tape configurations were designed using 3D printing technology: tapes with one twist, four twists, and four twists with perforations. Experiments were performed using a PVC pipe with a carbon steel plate as the material under investigation. Slurries of water and silica sand were prepared with varying sand concentrations—1%, 3%, and 5%—to induce different erosion rates. The experimental results were backed by Computational Fluid Dynamics (CFD) using the discrete phase model (DPM) to predict particle flow and erosion attributes. Erosion trends were also tested through mass loss and paint loss tests. The analysis outcomes demonstrated that the one-twist, four-twist, and perforated four-twist tapes reduced the erosion rate by 18%, 39%, and 45%, respectively. Among the different configurations, the four-twist tape with holes reduced erosion the most. These results suggest that twisted tape inserts can control erosion, thereby increasing the service life of pipes that handle abrasive flows.

## 1. Introduction

The ASTM International Standard G40-13 defines erosion as the gradual loss of material from a solid surface as a result of the transport and impact of fluids, multiphase streams, droplets, or solids [1]. Crook classified erosion wear into four types—namely, SPIE, slurry erosion, liquid droplet impingement erosion, and cavitation erosion—which are the most common types. Of these, solid particle impingement erosion occurs mostly in industrial systems, where it leads to wear of pipeline bends, feeders, cyclone separators, etc., which affects the reliability and safety of equipment, especially in pneumatic conveyor systems [2]. Sand—particularly small hard sand particles—is considered a major cause of pipeline erosion in hydrocarbon systems. When produced along with hydrocarbons, sand poses a risk to well construction and surface facilities [3]. Since about 7% of oil and gas resources include sand formation, actions that prevent early structural and pipeline abrasion are essential.

The literature on the effectiveness of twisted tape inserts is extensive. For example, Wood [4] observed that in pipeline bends, swirling flow greatly minimized erosion. Malka et al. [5] determined that turbulence originating from disturbed liquid particle flow increases erosion and corrosion. Cazan and Aidun [6] demonstrated that the secondary vortices caused by twisted tapes increase the tangential velocity at the wall, thereby diminishing the particle deposition tendency. Sarada et al. [7] showed that twisted tapes can help to enhance flow characteristics and mitigate erosion risks. Numerical and experimental methods have expanded the knowledge base on twisted tape inserts due to research innovations. In 2018, Ossai and Arsha [8,9] further demonstrated that twisted tapes limit localized effects by distributing particles in elbow bends. Zhou and Santos [10,11,12].

Another area of concern is the proper design of the twisted tape’s geometry. Yadawa and Zhou [13,14] studied twist ratios to achieve high performance. In their work, Al-Obaidi [15] showed that twisted tapes with variable pitch yielded a higher heat transfer coefficient than those with constant pitch. Paneliya [16] demonstrated how segmented twisted tapes improve the local heat transfer and minimize erosion, and Liu and Tusar [17,18] investigated the effects of the geometry of perforated twisted tapes on erosion and heat transfer [19].

Kaliakatsos et al. conducted a CFD analysis of pipes with twisted tape inserts, where the twist ratio was altered. According to their results, the convective heat transfer coefficient rose by 30% (L/D = 16) to 81% (L/D = 2) when using crude oil as the working fluid. Lower twist ratios favored heat transfer but were associated with higher pressure drops, thus emphasizing the need to optimize heat transfer and pressure losses during design [20]. Further, in 2019, Wijayanta [21] studied internal flow in an enhanced tube with a square-cut twisted tape insert. They reported that the flow is fully turbulent at low Reynolds numbers with a reduction in flow length.

Many studies on erosion protection have employed CFD models, with few practical applications, thus offering minimal insight into actual erosion occurrences. Previous works analyzed only predetermined twisted tape geometries without seeking to improve them or studying how sand concentration affects thickness loss and hardness. Earlier research on erosion-control inserts has reported reductions of 10–40%, depending on the insert geometry, flow regime, and particle characteristics.

The objective of this study is to evaluate the impact of three twisted tape configurations (one twist, four twists, and four twists with holes) on the reduction in erosion at various solid–liquid ratios (1%, 3%, and 5%) compared to an untwisted pipe. Particle impact angle, near-wall turbulence, and boundary-layer disturbances affect erosion caused by slurry flow. Due to cutting and plowing, ductile materials like steel usually experience maximum erosion at low impact angles (20–30°). Twisted tape inserts lower the overall erosion rate by changing the flow path and decreasing the frequency of these low-angle particle collisions. This study’s goal is to improve twisted tape inserts, with a focus on minimizing particle contact effects, enhancing pipeline durability, and innovating new strategies for erosion management in the oil and gas sector, as well as in chemical industries that use abrasive slurry flows.

Several CFD studies have examined erosion phenomena in liquid–solid and gas–solid flows, mainly addressing particle trajectory prediction, wall shear stress distribution, and target areas of pipelines subject to erosion. Numerical studies [2,3] have shown that flow distortions (secondary swirling flow, recirculation zones, and velocity gradients) are important factors in increasing material loss rates in elbows, contractions, and straight pipes. Although many researchers have modeled erosion in Eulerian–Lagrangian frameworks combined with discrete particle tracking (DPM), which considers internal flow devices such as twisted tape inserts to mitigate erosion, the literature appears to be scarce.

Past studies on twisted tape inserts have focused more on improving the performance of the heat transfer operation. Several studies have examined conventional twisted tapes, perforated tapes, and tapes with multiple twists with the use of thermofluids. Nonetheless, the literature demonstrates limited examination of twisted tapes as end-of-life erosion-reducing devices in multiphase liquid–solid flow. In addition, the majority of commercially available designs are produced using traditional fabrication methods, limiting the flexibility of their geometries and prohibiting the formation of intricate or optimal twist profiles. Conversely, the current work proposes three new 3D-printed twisted tape designs: single-twist, four-twist, and perforated four-twist inserts. These inserts are specifically employed to alter the contact between particles and walls and minimize erosive wear within the pipe. The introduction of additive manufacturing allows the production of precise, customizable, and repeatable twist geometries, thus substantially improving previously reported designs. This method provides new information on the hydrodynamic and particle-tracking processes that contribute to the observed decrease in erosion.

## 2. Materials and Methods

### 2.1. Three-Dimensional Printing of Twisted Tapes

**Figure 1:**
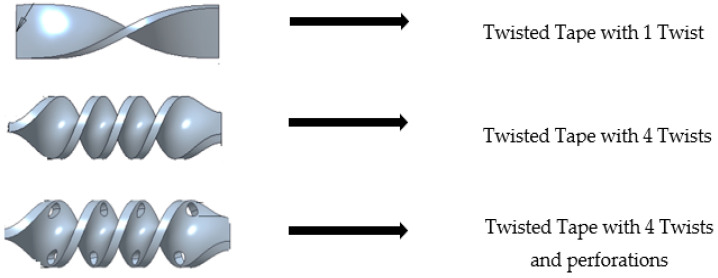
Various twisted tape geometries used in this study [22].

In this work, we utilized three different twisted tape inserts crafted from PLA. These twisted tapes were carefully modeled in the Creo Parametric software 5.0 and then 3D-printed, as illustrated in Figure 1.

The experimental test pipe was a commercial PVC pipe with an inside diameter of 17.5 mm, and carbon steel ANSI 1040 material, used for API pipes, was the target surface, with dimensions of 38.1 × 38.1 mm and a thickness of 2.5 mm. The composition of the carbon steel (AISI 1040) is shown in Table 1.

To guarantee a consistent surface texture, the sample surfaces were polished on a cotton wheel at 1500 rpm after being prepared with a flap wheel sander, using sandpaper with 400, 600, 800, and 1200 grit sizes. Samples were then degreased in ethanol, dried, and placed in desiccators to avoid exposure to moisture before the tests. For multilayer paint modeling, two layers of paint were applied: red followed by yellow. Using a grinder wheel, rust and impurities were scraped off; the area was further sanded and polished to achieve a smooth texture. In the experiment, we utilized slurry mixtures with three concentrations for testing. The tank’s total capacity was 30 L, and the amount of sand added to the tank was measured using a weight scale. For a 1% concentration, 0.3% (or 300 g) sand was added to the 30 L water tank; for a 3% concentration, 0.9% (or 900 g) sand was added to the tank; and lastly, for a 5% concentration, 1500 g of sand was added to the tank. All measurements of sand were accurately taken using a sensitive digital measuring scale (resolution ±0.01 g) before it was added to the water tank for the experiments.

**Figure 2:**
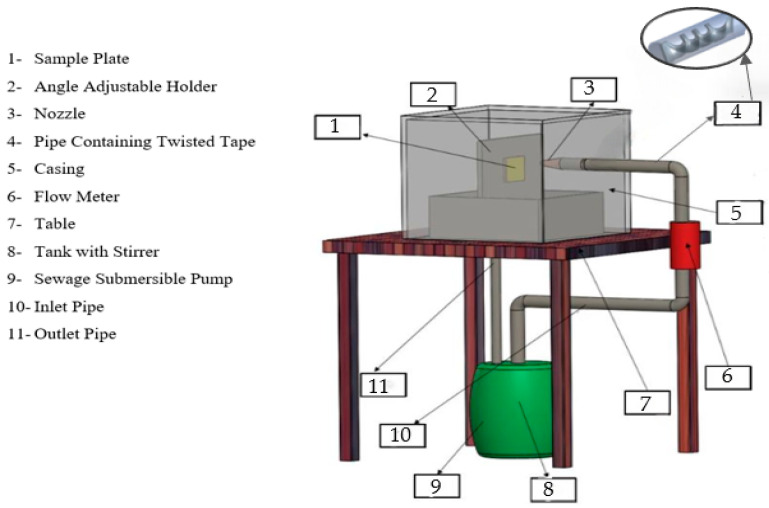
Experimental setup [22].

Figure 2 depicts the apparatus utilized in the experiments.

The tank contained the slurry mixture of sand and water, which was pumped through a pipe with a twisted tape insert and impacted a specimen plate. Tests lasted for 15 min for painted specimens and 8 h for polished specimens. The sample was put in an adjustable holder, and the slurry flowed through a pipe with a flow meter, nozzle, and twisted tape insert. Three pipes with different twisted tape helices were employed. All the erosion tests were performed at an impact angle of 90°, which provided the maximum force per particle on the sample. The flow travels in a closed circuit, thus reducing the loss of fluid as well as its fluctuation. The flow rate was continuously monitored with a turbine flow meter (accuracy ±1%), and particle uniformity was controlled with the help of mechanical stirring. Every experiment was replicated three times to ensure repeatability.

Preliminary erosion experiments were conducted using a PVC pipe because it is cheap and easy to manipulate and poses no problems as a controlled baseline for studying the behavior of slurry flows. The AISI 1040 carbon steel material was included because it is commonly used for industrial pipes, and its mechanical and erosion properties are well established. In this research, the term “plain pipe” is used to describe the part of the pipe with no twisted tape insert.

#### 2.1.1. Numerical Simulation and Computational Mesh

The Eulerian–Lagrangian scheme models water–sand mixtures by employing the Eulerian method to simulate fluid dynamics on a fixed grid and by applying the Lagrangian method to track dispersed phases via the discrete phase model. The method is very effective in capturing fluid–particle interactions, but it requires a huge amount of computation. For the continuous phase, hydrodynamic analysis is applied based on the Navier–Stokes equations and the k-epsilon model to observe fluid characteristics such as velocity, pressure, and turbulence, with contemplation of the system’s continuity and momentum.

(1) $$\partial \rho /\partial t\;+\;\nabla (\rho u^{\to })\;=\;0$$

(2) $$\partial (\rho u^{\to \to })/\partial t\;+\;\nabla (\rho u^{\to }u^{\to })\;=\;-\nabla \rho \;+\;\nabla (\overset{=}{\tau })\;+\;\rho gi\;+\;S_{D}$$

In Equations (1) and (2), the symbol *ρ* represents the density of the fluid, and $u^{\to }$ refers to the velocity vector of the fluid. The term p denotes the static pressure, while $\overset{=}{\tau }$ signifies the stress tensor, and μ stands for fluid viscosity. Additionally, *gi* represents gravitational acceleration, and *S*_*D*_ accounts for the additional source term resulting from interaction with the other phase.

Discrete phase modeling (DPM) is used to model the motion and behavior of particles, droplets, or bubbles in a continuous phase to assess the interactions in different flow systems. Particle trajectories are determined from forces such as drag, gravity, buoyancy, pressure gradient forces, and thermo-phoretic effects, together with continuous phase computations. The governing equation for DPM is

(3) $$m_{p}\frac{dup}{dt}=F_{D}+F_{G}+F_{VM}+F_{P}$$

After determining the particle mass *m*_p_, we consider the drag force *F*_d_, buoyancy force *F*_G_, virtual mass force *F*_VM_, and pressure gradient force *F*_P_. CFD-DPM simulations have been conducted.

This steady and unsteady interaction determines the turbulence of the fluid state. At high Reynolds numbers or velocity gradients, a fluid is considered turbulent, thus influencing momentum, heat, mass, and energy transfer. The RSM was chosen, as it solves the individual Reynolds stress components and can capture the anisotropic turbulence created by the twisted tape inserts, which the standard k-e model cannot.

The equations are expressed as follows:

(4) $$1\;-\;\frac{\partial \left(\rho \;u_{i}^{\prime }u_{j}^{\prime }\right)}{\partial t}\;+\frac{\partial \left(\rho \;U_{k}u_{i}^{\prime }u_{j}^{\prime }\right)}{\partial x_{k}}\;=\;P_{ij}\;+\;\Phi_{ij}\;-\;\varepsilon_{ij}\;+\;D_{ij}^{t}\;+\;D_{ij}^{\mu }$$

Terms:

$P_{ij}$ = $\left(\rho u_{i}^{\prime }u_{j}^{\prime }\right)$

$\partial x_{k}$ + $(\rho U_{k}u_{i}^{\prime }u_{j}^{\prime }$)—Production term;

$\Phi_{ij}$—Pressure–strain correlation;

$\varepsilon_{ij}$ = Dissipation tensor;

$D_{ij}^{t}$—Turbulent diffusion;

$D_{ij}^{\mu }$—Molecular diffusion.

#### 2.1.2. Dissipation Rate Transport Equation


(5) $$\frac{\partial \left(\rho \;\varepsilon \right)}{\partial t}+{\frac{\partial \left(\rho \;U_{k}\varepsilon \right)}{\partial x}}_{k}=C_{\varepsilon 1}\left(\frac{\varepsilon }{k}\right)P_{k}-C_{\varepsilon 2}\rho \frac{\varepsilon^{2}}{k}+\frac{\partial }{\partial x}\_k\;(\left(\mu +\frac{\mu_{t}}{\sigma_{\varepsilon }}\right)\frac{\partial \varepsilon }{\partial x}\_k)$$

Terms:

k = Turbulent kinetic energy;

$P_{k}$ = Turbulence production;

$\mu_{t}$ = Turbulent viscosity;

$C_{\varepsilon 1},\;C_{\varepsilon 2}\;$= Model constants.

The term *S*_*ij*_ represents the turbulent strain rate tensor, and *E*_*ij*_ is the mean rotation rate tensor in Equation (4). In Equation (5), the symbol ϵ represents the turbulent dissipation rate, while *ω* stands for the specific dissipation rate.

Abrasives are likely to erode any equipment in the flow path, such as valves and pumps. The Oka [23,24], Arabnejad [25], and Generic models [26,27,28] are helpful in their own ways for certain erosion forms. No model is free from error, but the Oka model has received significant acceptance and correlates best with empirical estimates. This model is semi-empirical and considers various parameters: the velocity and angle of impact, particle size, and the material of the particle and the target; therefore, it can be used to predict erosion rates in various situations. The model is defined by the following equation:

(6) $$E\;=\;E0\;{\left(\frac{v_{p}}{v_{ref}}\right)}^{k_{2}}\left(k_{2}\right)\;{\left(\frac{D_{p}}{D_{ref}}\right)}^{k_{3}}\;f\left(\theta \right)$$

where V_p_ is the impact velocity of the particle, v_ref_ the impact velocity of the reference, D_p_ the diameter of the particle, and D_ref_ the diameter of the reference; k_2_ and k_3_ are constants. At the impact angle, v_p_ = v_ref_. In Equation (6), f denotes the impact angle.

### 2.2. CFD Modeling

**Figure 3:**
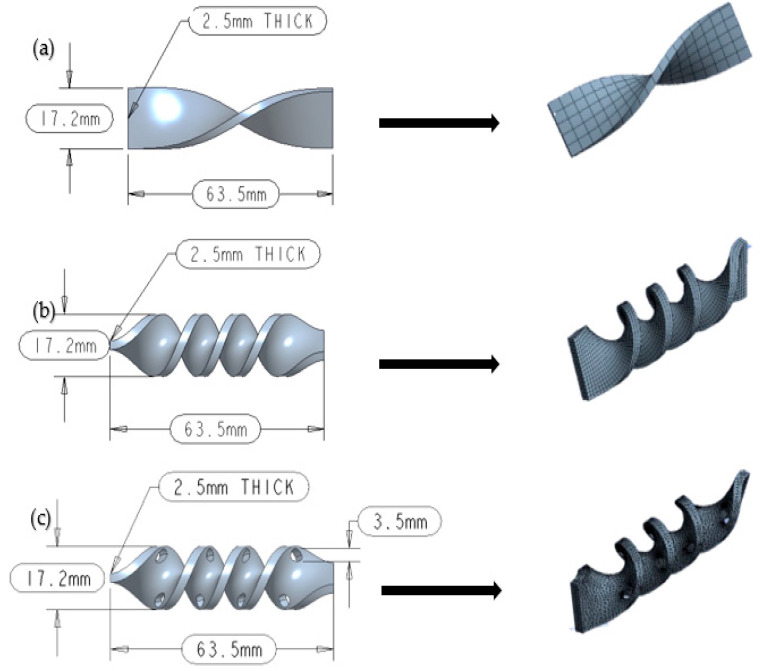
Geometric configurations and meshing: (**a**) 1-twist tape, (**b**) 4-twist tape, (**c**) 4-twist tape with perforations [22].

A sand–water flow environment simulation of PLA (Polylactic Acid) twisted tape was performed using CFD-DPM. The twisted tape geometries are shown in Figure 3, with each twisted tape being 63.5 mm (2.5 inches) long, 2.5 mm (0.0984 inches) thick, and 17.2 mm (0.677 inches) in diameter, as shown in Figure 3.

After designing these geometries, we used a fine meshing technique to assess the outcomes. Three grid densities, coarse, medium, and fine, were used to perform a mesh independence study. The number of elements was gradually increased, and the pressure drop, velocity distribution, and erosion rate were compared. The difference in erosion rate between the medium and fine meshes was less than 3, indicating convergence of the mesh. Thus, the final simulations were conducted using the medium mesh because it offered a good balance between accuracy and computational cost. To create the mesh and obtain computational results, we used the ANSYS software (2020-R1). Figure 3 shows the meshing of each twisted tape.

The twisted tape inside the pipe is inserted in a manner that allows it to be securely fixed to the pipe walls. Improper fitting may prevent the fluid from exhibiting a swirling motion, as the twisted tape inside the pipe may rotate due to the flow.

**Figure 4:**
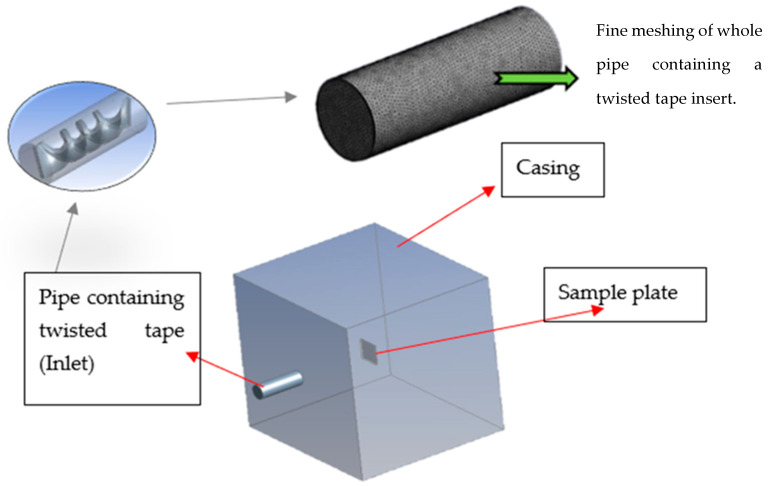
CFD experimental setup [22].

The experimental setup for CFD analysis is depicted in Figure 4.

The pipe with twisted tape inserts serves as an inlet for the slurry mixture, and the test plate is positioned in the path of the flowing fluid. After passing through the pipe with the twisted tape insert, the fluid strikes the sample plate and creates impressions due to erosion. The outer casing functions as an outlet to complete the CFD setup. This entire experimental setup was designed in ANSYS.

Table 2 shows the simulation parameters used to obtain the CFD results.

The boundary conditions of the simulation were as follows: an inlet velocity (5 m/s) was imposed at the entrance of the pipes, and a pressure outlet condition was imposed at the pipe exit. No-slip boundaries were assumed for all pipe walls. In the erosion simulation, the DPM was applied with specified particle characteristics and restitution coefficients. The casting walls were taken to be adiabatic, and no further thermal loading was taken into account because flow-induced erosion was considered.

The cases used to conduct the experiments are summarized in Table 3. Twenty-four experiments were conducted. Painted specimens were used in 12 experiments, while polished test samples were used in the remaining 12.

The experiment ran for 15 min to produce and visualize the results. However, when this duration was used for the polished samples, no erosion was observed. Given that erosion is a slow process, the duration of the experiment was extended from 15 min to 8 h. Subsequently, the experiment continued for 8 h, and erosion of the samples was observed. The sand particles had an average size of 50–60 µm, and the flow velocity was kept constant at 5 m/s. Profilometry was performed on dried samples that had been cleaned with ethanol. A microscopic analysis was adopted to distinguish wear pits and surface roughness or printing marks in terms of a threshold.

## 3. Results and Discussion

### 3.1. Multilayer Paint Modeling

**Figure 5:**
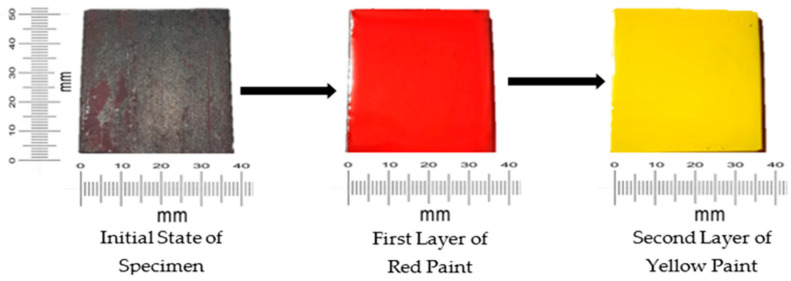
Steps for preparing sample for multilayer paint modeling [22].

We used a multilayer paint test to examine the qualitative behavior of paint erosion in the context of liquid–solid flow. Using a digital coating thickness gauge, we applied two layers of paint—red and yellow—to the surface of the sample plate, each with a thickness of 45–50 µm. As shown in Figure 5, this enabled us to identify patterns of erosion hotspots.

The paint layering shows where the sample plate was most affected by two-phase flow erosion. It pinpoints the area that was hit the hardest by the particles. The test was performed with a water velocity of 5 m/s, a particle size of 50–60 µm, and a sand concentration of 1%. Paint removal was the highest without twisted tape, while it was slightly reduced by one or four twists. The erosion rate was lower when using higher twist ratios and tapes with holes, indicating that a higher twist and hole count leads to less erosion than a lower twist count. Similar results were reported by Arsha [9] and R. Khan et al. [29].

**Figure 6:**
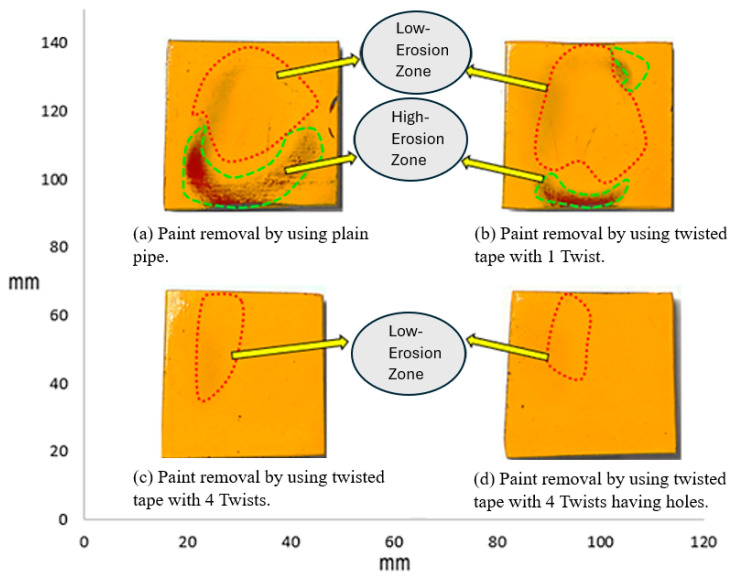
Paint erosion patterns for erosive slurry flow with 1 wt.% sand fine concentration [22].

Figure 6 shows the erosion pattern on the sample plate impacted by 1 wt.% sand concentration. The plain pipe caused the highest paint loss as a result of flow impact. The one-twist tape reduced paint removal from the chips by scattering the particles evenly. The four-twist tape surpassed this result and further reduced paint removal by increasing flow swirl. The least paint was removed when using the four-twist tape with holes because the holes added turbulence, which resulted in the most balanced distribution of particles.

**Figure 7:**
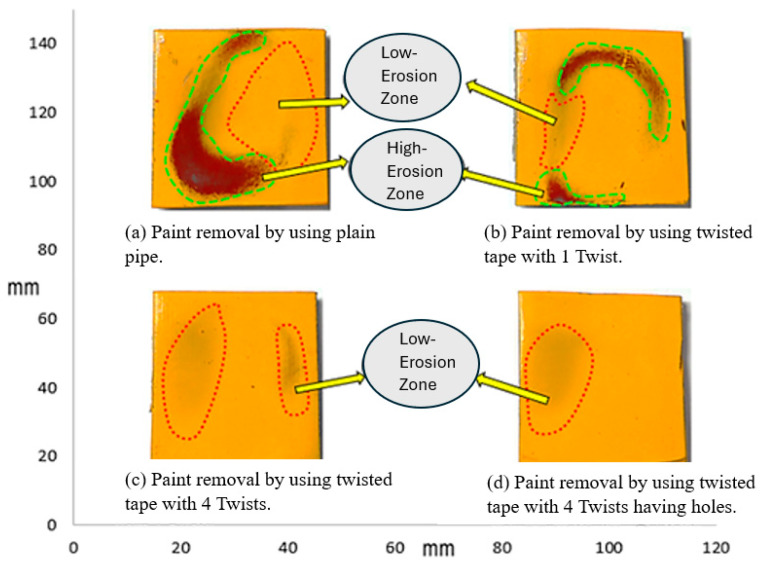
Paint erosion pattern for erosive slurry flow with 3 wt.% sand fine concentration [22].

**Figure 8:**
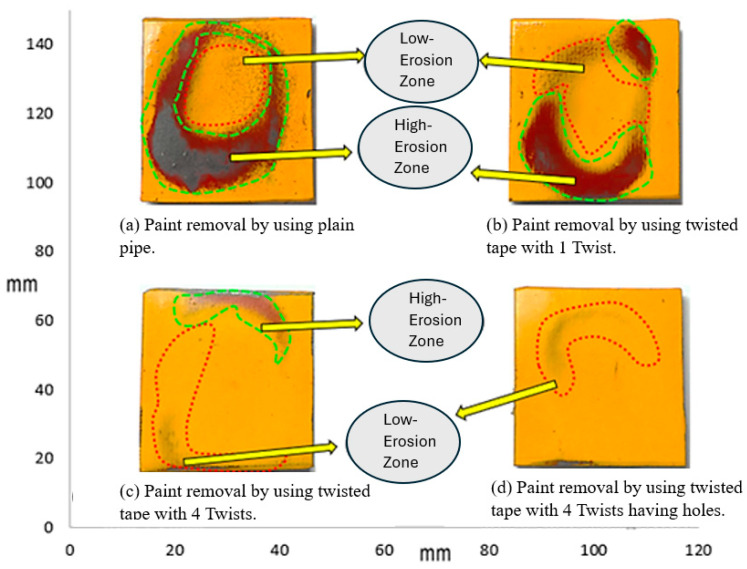
Paint erosion pattern for erosive slurry flow with 5 wt.% sand fine concentration [22].

Figure 7 demonstrates the pattern of erosion experienced by the sample plate impacted by a 3 wt.% sand concentration. The higher sand concentration led to enhanced paint stripping because more particles contacted the surface. The erosion rate was thus higher than that observed with the 1% sand concentration. In the plain pipe (a), high- and low-erosion zones were evident, and the maximum paint loss was due to direct particle impact. Compared to the smooth pipe, the one-twist pipe (b) exhibited a slightly lower degree of erosion, and the four-twist pipe (c) prevented the flow of particles, thus minimizing erosion. The perforated four-twist pipe (d) had the smallest percent paint loss and provided the most protection from the particles. Increasing the sand concentration to 5% further increased paint removal, as shown in Figure 8.

After looking at the three cases tested with different amounts of sand (1%, 3%, and 5%) to see how quickly paint was worn away, we found that more sand in the mix means faster paint wear. Therefore, a fluid with a lot of sand will erode paint more quickly than one with just a little. This finding was also reported in a previous paper [30]. In addition, the paint wore away in noticeable circular patterns.

### 3.2. Mass Loss Analysis

**Figure 9:**
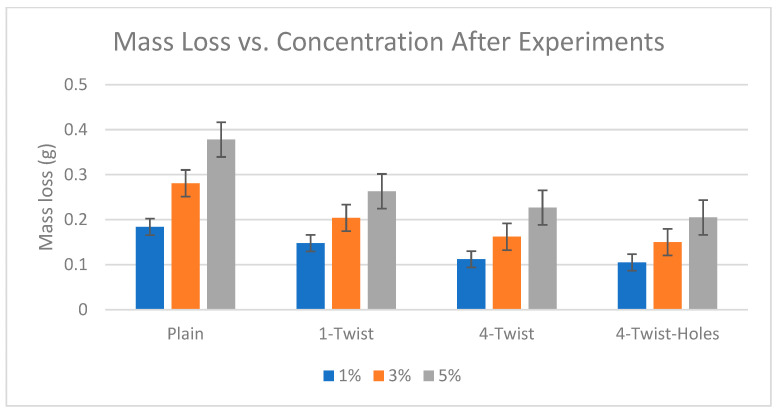
Mass loss of sample plate due to erosion [22].

Mass loss analysis was also performed by R Khan [29,31], who used different sand concentrations in two-phase flows. Figure 9 shows the total mass of eroded particles collected from the sample plate with a polished surface in the water–sand two-phase flow. The sand percentage ranged from 1 to 5 percent. In these experiments, the mass loss was significantly higher with the one-twist tape and the lowest with the four-twist tape with center holes. The twisted tape inserts alter the internal flow to create swirl and secondary vortices, which carry the particles to the center of the flow, where they are less likely to interact with the inside of the pipe. This swirling action reduces the normal component of particle impact velocity and removes particles at shallow impact angles (20–30°), which normally lead to maximum erosion in ductile materials such as carbon steel. Consequently, the rate and intensity of particle–wall collisions are reduced, resulting in a significant decrease in erosion, especially in perforated four-twist tapes. The sample plate mass loss was weighed on a precision balance before and after the experiment, which was performed at a constant flow velocity of 5 m/s with sand concentrations of 1%, 3%, and 5% and sand particle sizes between 50 and 60 µm.

Table 4 shows the sample plate masses before and after the experiments and the calculated mass losses. Before measuring, we thoroughly dried the sample plate to prevent moisture from affecting the weight. We measured the masses with a precision scale in a closed, controlled space to keep air and other particles from changing the sample plate’s original mass.

The results indicate that the sedimentation rate was the highest at 5% sand concentration and the lowest at 1% because of more abrasive particles altering the surface impact. These results are consistent with the literature [32,33,34]. One can observe that, among all samples, the four-hole samples had the minimum mass loss, because the applied four-twist tape design forced the fluid to rotate, thus counteracting opposing streams.

### 3.3. Thickness Loss Analysis

The reduction in thickness was measured to identify the erosion profile and surface damage on the plate subject to fluid strikes. In contrast to thickness measurements in weight loss, thickness loss tests can indicate a specific stressed or impacted area, which is very valuable for inhomogeneous materials or geometries. This technique offers complementary information about erosion processes—such data are crucial in areas such as pipelines, where wall losses should be minimized. By pinpointing particular wear zones, thickness loss tests enable better control of erosion in regions that have been determined to be most critical due to wear.

Surface roughness was added to the CFD model, in that all wall boundaries were assigned the roughness value measured on the pipe material. The near-wall region was resolved using standard wall functions, and the mesh was refined to ensure that the y+ values were within the recommended range used in erosion modeling. The near-wall y+ values in this study ranged from 50 to 180, which guaranteed proper prediction of particle–wall interactions, as well as the erosion rate.

**Figure 10:**
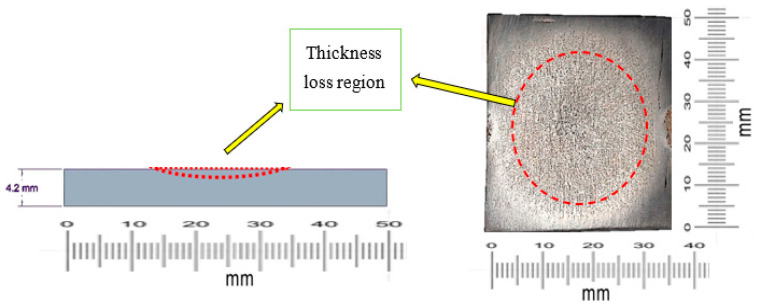
Thickness loss region of the target plate [22].

We started with a target material that was 4.200 mm thick. When the fluid hit the center of the plate, we noticed material loss there, as can be seen in Figure 10.

We used a precise screw gauge to measure the target plate’s thickness before and after the experiment. Table 5 shows the thickness loss data.

**Figure 11:**
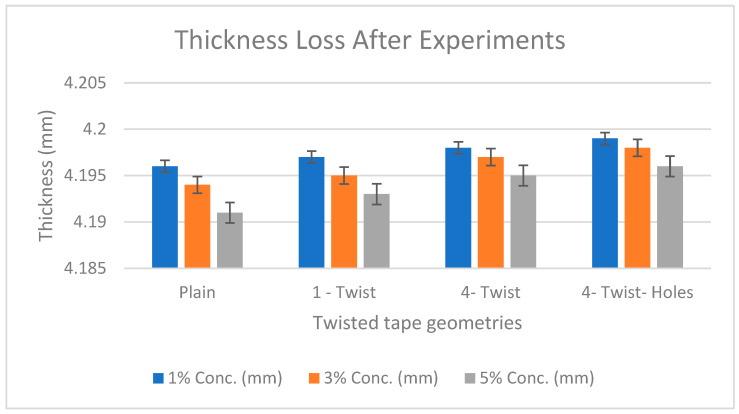
Thickness loss chart [22].

Velocities exceeding a certain threshold increase the erosion rate, depending on the angle of fluid attack on the eroded surface. Erosion is best tackled by harder grades of equipment than softer grades, and particulates or corrosive fluids cause faster material loss. In the thickness loss analysis, the pipe with a four-twist tape with perforations, used to trap particles, experienced less thickness reduction than the other two pipes, as the particles move in circular movements across the pipe’s surface, hence reducing the thickness loss rate due to erosion as shown in the Figure 11.

### 3.4. Hardness Test Analysis

Hardness is very closely related to the rate of material erosion; in this case, harder materials are less prone to erosion because they experience minimal plastic deformation and general surface wear. However, the erosion rate also varies with such factors as the erosion mode (ductile or brittle), impacts, and the characteristics of the particulate matter—size, hardness, and velocity. However, the overall trend is that hardness increases are associated with erosion declines, although variation in this trend in different erosive contexts cannot be entirely ruled out [29,35].

The hardness of the samples was analyzed using the Vickers hardness method. We used a square, pointed diamond to make indents in each sample at three spots. The obtained hardness values are given in Table 6.

Accordingly, the data highlight that plain tapes are the hardest of all the samples and are therefore most likely to have the highest wear or erosion resistance. The plain tape’s hardness varies from 84 HV at 1% concentration to 87.77 HV at 5% concentration, attributable to enhanced crystallite size and dislocation density. The HS of the one-twist tape is lower, lying in the range from 81,48 HV at 1% to 85 HV at 5%, meaning that while one twist decreases HS, the higher concentration increases it. The four-twist tape exhibits higher elongation than the two-twist tape at each concentration, with 78 HV at 1% and rising to 83 HV at 5%, demonstrating increasing hardness with higher concentration. The four-twist tape with holes offers the least in hardness, ranging from 78.11 HV at 1% to 82.89 HV at 5%; holes in the material reduce its hardness and ability to resist indentation.

**Figure 12:**
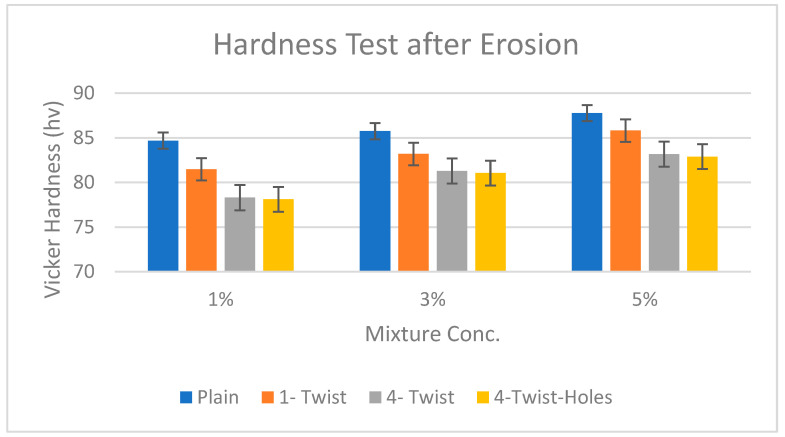
Hardness test chart [22].

The above table and description can be best visualized by the bar chart shown in Figure 12.

The bar chart shows the Vickers hardness at the three concentrations (1%, 3%, and 5%) using twisted tapes with different configurations. All the “plain pipes” had higher hardness than the “one-twist tapes” at all concentrations. The “four-twist” and “four-twist with holes” produced the least hardness among all the tested samples. In a previous study, the hardness of a concrete mixture was found to rise with increased mixture concentration because of strain induced by the kinetic energy of the abrasive and fluid particles implanted in the concrete sample; the implication was that higher concentration translates to higher material hardness [34].

### 3.5. SEM Analysis

Scanning electron microscopy (SEM) is a method for examining material structures at the nanometer scale using secondary electron imaging. As SEM employs a focused electron beam to scan the sample surface, it produces high-resolution images. When electrons interact with the atoms on the surface, various signals are generated and converted into images. SEM is used to study surface morphology, such as crack patterns, pits, particle size and shape distribution, and the composition of materials, including metals, polymers, ceramics, and biological samples.

Pitting refers to small, localized structures resulting from particle impacts, while plowing describes elongated grooves formed by sliding particles that dislodge material on the sides. Macro perforations, which are deep, large structures of damage due to severe or repeated impacts, cause significant material removal.

**Figure 13:**
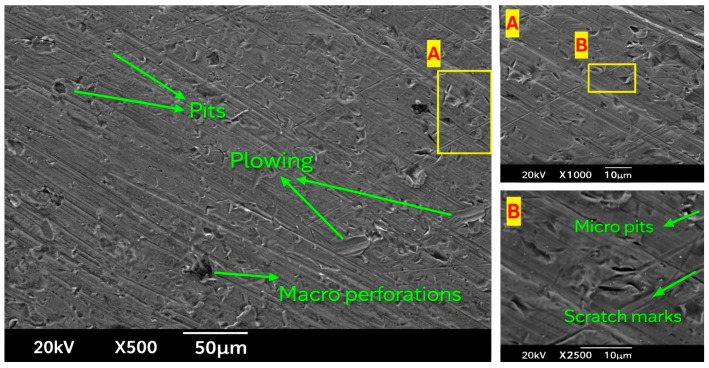
SEM analysis of a sample at 1% sand conc. and with the plain pipe [22].

Image 1 (Left)—500× Magnification: Small pits show that material has been removed locally due to sand particle impacts. Various features such as plowing in the Figure 13 indicate that abrasive particles roll over and abrade the surface by depositing material onto another substrate. Macroscopic dimensions associated with large pellets can substantially decrease the thickness of the material layer and determine the degree of wear. Image 2 (Top Right)—1000× Magnification: Focus Area ‘A’ reveals even more detail, with diverse pits and grooves, and therefore provides a better understanding of the erosion processes—for instance, the angle of the impact based on the pit’s shape. Image 3 (Bottom Right)—2500× Magnification: Focus Area ‘B’: Micro pits and scratch marks are identified in Focus Area ‘B’. Micro pits, shorter and less dense, indicate that the smaller or lower-energy particles are capable of cutting metal, while scratches indicate the direction and area of particle flow, as well as the hardness of the material.

**Figure 14:**
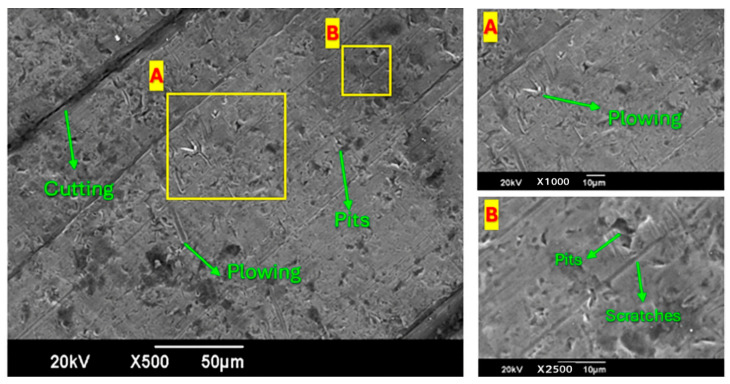
SEM analysis of a sample at 5% sand conc. and with twisted tape having 1 twist [22].

SEM images of the sample plate with 1% concentration and twisted tape (one twist) are shown in Figure 14. The results show less erosion than that in the prior SEM images (Figure 14), which can be attributed to the use of the one-twist tape. Below is a detailed explanation of each image in the context of erosion due to sand particles. Image 1 (Left)—500× Magnification: Bare surfaces are discernible and show that sand particles possessed enough energy to carve crevices into the organic matter, but the level of erosion is lower than in previous pictures. There is plowing, but it is somewhat diminished compared to previous samples, again implying that the forces are weaker, and the material is not transported as vigorously owing to its whirlpools. Fewer and shallower holes represent less erosion of the ground surface. Image 2 (Top Right)—1000× Magnification: Fewer and shallower ridges can be seen in Focus Area ‘A’, which adds to the evidence that less erosion occurs due to either less force or fewer abrasive agents being applied. Image 3 (Bottom Right)—2500× Magnification: Focus Area ‘B’: Finer particles in pits and shallower scratches can be observed in Focus Area ‘B’, implying moderate erosion and generally low material removal. These results are in accordance with the literature [34,36].

**Figure 15:**
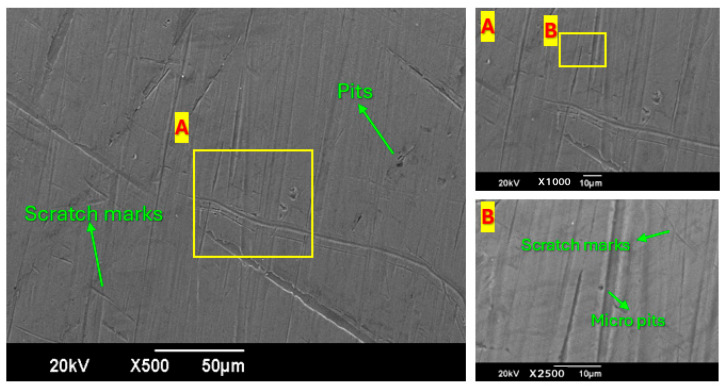
SEM analysis of a sample at 1% sand conc. and with 4-twist tape [22].

The SEM images in Figure 15 show the surface of the sheared and eroded material. The samples were tested with a system using a four-twist tape to increase the fluid’s swirling motion. Prior studies suggest that this swirling motion weakens the direct effect of sand particles on the sample, reducing the erosion rate compared to the earlier SEM images. Each image is described below.

Image 1 (Left)—500× Magnification: There are scratch marks, but they are not as deep or numerous as they were on the plate without the tape. This reduction occurs because the fluid motion created by the twisted tape dislodges sand particles into the flow and reduces their interaction with the surface. The scratches are few and small, supporting the theory that the surface is barely eroded. Image 2 (Top Right)—1000× Magnification: In Focus Area ‘A’, a few small and shallow pits are evidenced, which indicates less erosion. This should reduce surface wear, likely because the twisted tape has folds that assist in deflecting particles. Image 3 (Bottom Right)—2500× Magnification: The regions of Focus Area ‘B’ reveal fewer and less marked scratches and micro pits, suggesting that the swirling flow had a gentle wear effect on the surface, which lessened the sand impact.

### 3.6. Numerical Simulation

#### 3.6.1. CFD of Plain Pipe with No Twisted Tape Insert

In this section, we describe CFD-DPM simulations conducted for two-phase (liquid–solid) flow with particle sizes ranging from 50 to 60 µm. The modulation of turbulence by a similar swirl and the redirection of particle paths by this swirl have been extensively documented in CFD and experimental codes of slurry and multiphase pipe flows [20]. Such experiments prove that swirl-generating devices modify the axial and tangential velocity components, prevent the concentration of particles near the walls, and divert the impact angle of the particles to avoid erosion-prone areas. This reduces the particle’s kinetic energy at the wall and results in less intense and more uniformly distributed wear. The simulations were conducted in a pipe without a twisted tape insert. The slurry mixture flows through the plain pipe and impinges directly on the test plate.

**Figure 16:**
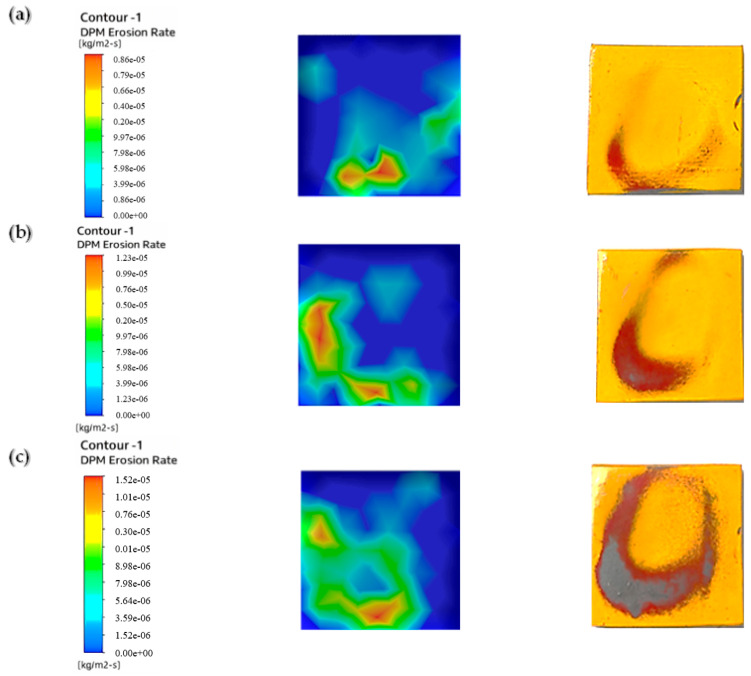
CFD results for plain pipe at (**a**) 1% conc., (**b**) 3% conc., (**c**) 5% conc [22].

Figure 16 shows how the sample plate was eroded under flow in the plain pipe with slurry mixes of 1%, 3%, and 5%.

The figure illustrates that at this location, the erosion rate rises with the concentration of abrasive particles in the slurry. The study using the CFD discrete phase model (CFD-DPM) showed that different cases yielded varying erosion profiles, with the 1% concentration having a lower erosion rate due to more water content and fewer sand grains. These results align with those of previous studies [37,38]. The pressure values obtained from CFD-DPM closely match experimental values, although there are slight differences in quantitative results, which may be due to aeration or neglecting spherical particle effects and particle–particle rebound in the model. Table 7 provides the erosion rate results from CFD-DPM and their variance from the experimental readings.

#### 3.6.2. CFD of Plain Pipe with a One-Twist Tape

**Figure 17:**
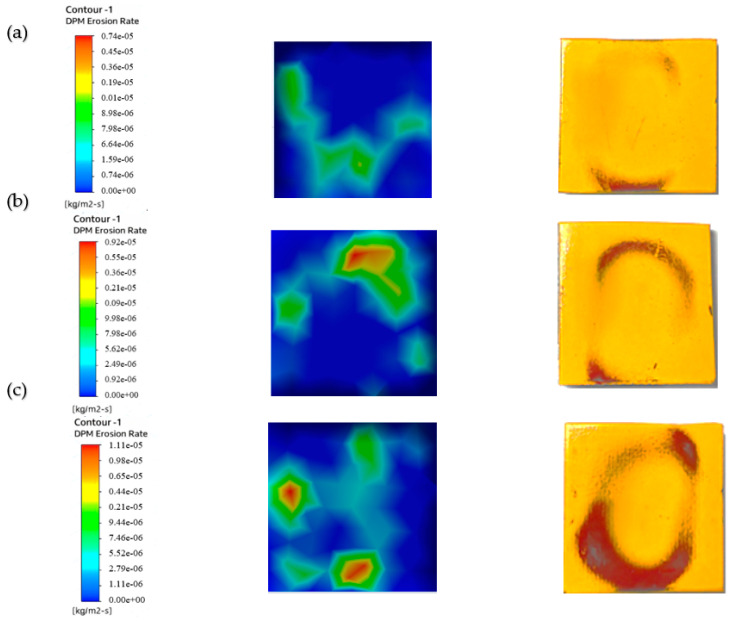
CFD results for tapes with 1 twist at (**a**) 1% conc., (**b**) 3% conc., (**c**) 5% conc [22].

To reduce erosion, a pipe with a one-twist tape was used. The slurry ran through this pipe and hit the test plate. Figure 17 shows the erosion on the plate caused by the slurry after it passed through the pipe with the twisted tape. Three slurry mixtures were used to create these erosion patterns: 1%, 3%, and 5%.

A significantly lower erosion rate compared to the CFD-DPM for the plain pipe flow shown in Figure 16. This swirling reduced direct impingement of the fluid on the test sample, thus minimizing erosion by the fluid. The sand used was abrasive and was applied in equal proportions across the sample plate, thus preventing wear from concentrating in specific regions on the plate. Therefore, as the sand concentration increased, the erosion rate increased because there were more abrasive hits. Table 8 compares the CFD-DPM erosion rates with the experimental values.

#### 3.6.3. CFD of Plain Pipe with a Four-Twist Tape

**Figure 18:**
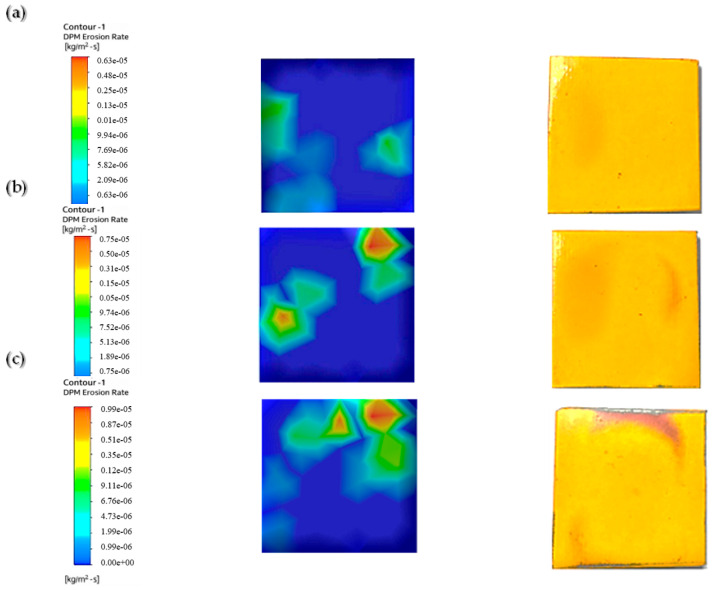
CFD results for 4-twist tape at (**a**) 1% conc., (**b**) 3% conc., (**c**) 5% conc [22].

Figure 18 shows erosion patterns on the sample plate. The patterns came from fluid that passed through a pipe with a four-twist tape insert. A slurry mixture with abrasive particles flowed through the pipe. We tested three mixture densities: 1%, 3%, and 5%.

Figure 18 depicts a much lower erosion rate compared with the CFD-DPM outcomes of the single-twist tape due to the augmented swirl from four twists. This enhanced swirl helped to substantially lessen direct flow impacts and spread abrasive particles evenly over the sample plate. At 1% sand concentration, the erosion rate was considerably lower than at higher concentrations, which have more striking particles and a weaker swirling motion, as mentioned before. These reductions increase the sample plate’s durability, as they minimize erosion. Table 9 shows the difference in results obtained from CFD and the experiments.

#### 3.6.4. CFD of Plain Pipe with a Four-Twist Tape Having Perforations

**Figure 19:**
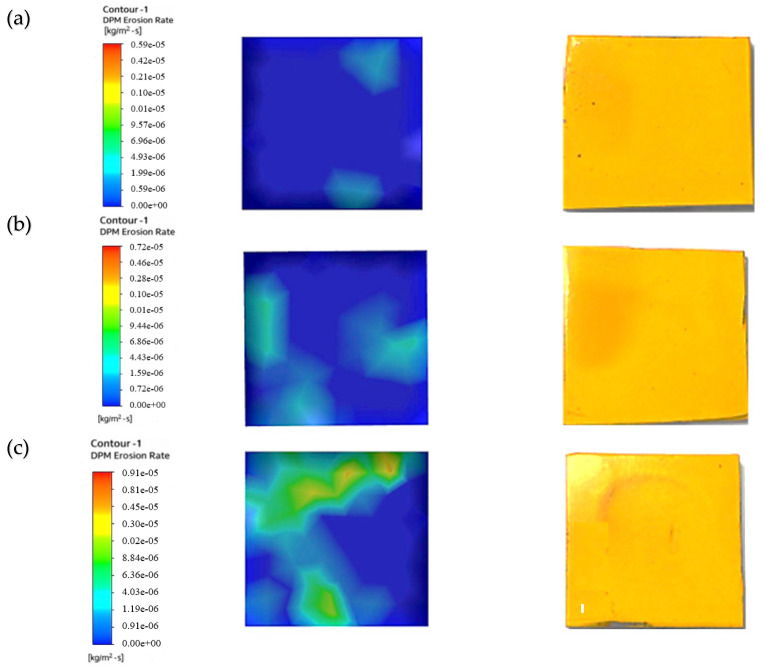
CFD results for 4-twist tape with holes at (**a**) 1% conc., (**b**) 3% conc., (**c**) 5% conc [22].

To further lower the erosion rate, a four-twist tape with perforated center holes was put inside the pipe. Figure 19 shows the erosion on the sample plate caused by the pipe with the twisted tape (four twists with holes). Three slurry mixture concentrations were tested: 1%, 3%, and 5%.

Figure 19 demonstrates a lower erosion rate compared with the four-twist tape; this additional improvement is due to the swirling and laminar movements of the fluid through the tape’s holes. This turbulence-inducing interaction reduced the rate of erosion to its lowest recorded level. The erosion rate was much lower at 1% sand concentration because, owing to the reduced number of sand particles striking the surface, the abrasive action of the fluid’s turbulence was also considerably reduced. Similar work was conducted previously [36]. Table 10 shows the erosion rates obtained from CFD-DPM and their difference from experimental readings.

#### 3.6.5. Comparison of Experimental and Numerical Erosion Rates

We found the erosion rate using both experiments and CFD-DPM simulations [35]. The results from the two methods were similar, though there were a few small differences. These may be because the simulations did not consider factors like the round shape of the particles, how particles bounce off each other, fluid temperature, or potential dirt in the sand. Including these details in the simulations might make the results match better. Table 11 compares the numbers from the experiments and CFD-DPM simulations.

The experimental results differ from the CFD-DPM values by about 0.051 g. Despite this difference, the CFD-DPM results are close to the experimental findings and can be considered reliable.

### 3.7. Particle Tracking

**Figure 20:**
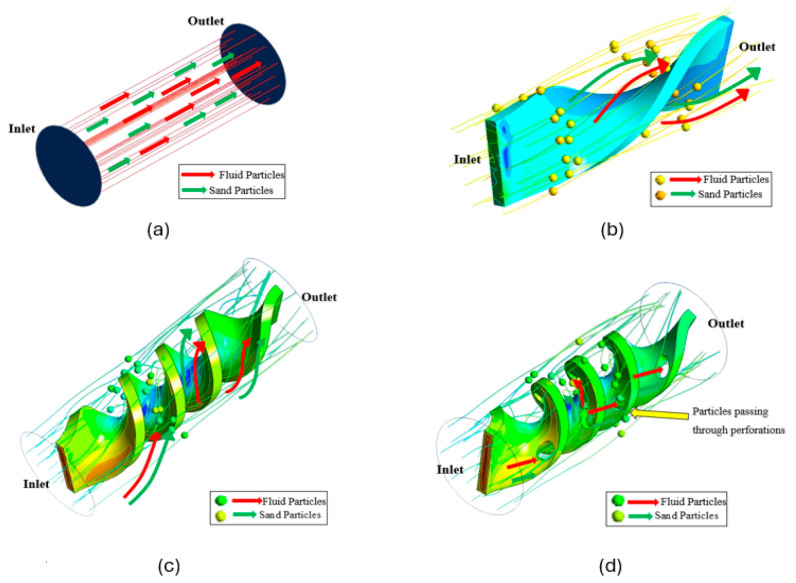
Particle tracking: (**a**) plain pipe, (**b**) 1 twist, (**c**) 4 twists, (**d**) 4 twists with perforations [22].

In a two-phase flow, particle speed follows a curve, hitting its highest point at the pipe’s center and dropping to zero at the wall because the fluid sticks to the surface. When a twisted tape is put inside the pipe, the flow swirls, becomes more chaotic, and spreads particles more uniformly across the plates, which boosts coverage. The twisted tape alters the motion and prevents sand particles from settling, leading to a steadier flow, as Figure 20 shows.

Particles tend to move in straight, parallel lines along the pipe, and therefore, they undergo very little mixing, which can cause serious erosion, as described in [37,38]. A twisted tape insert forms a helical geometry, which directs the fluid through a spiral path. This swirling motion improves mixing of the particle suspension and enhances the heat transfer rate. In the present experiments, sand and water transported through the pipe with four twists are affected by the twist ratio—the ratio of the length of the tape per turn to the pipe internal diameter. When the twist ratio decreases, precession and turbulence are enhanced, with improved sand–water mixing that minimizes particle settlement. The twisted tape with perforations improves turbulence and provides nearly equal distribution of particles, which decreases erosion compared with other layouts. The flow dynamics factor varies with the tape structure, hole size, particle size, and nature of the fluid; tapes are turbulent and thus do not allow considerable deposition, but details can differ based on the flow velocity and the size of the particles.

In summary, using a twisted tape with centered perforations in the pipe creates an intricate flow pattern. Water and sand swirl, creating turbulence and secondary flow. This boosts suspension and circulation of the particles, which decreases erosion compared to other methods mentioned.

## 4. Conclusions

The objective of this flow investigation was to determine whether 3D-printed twisted tape inserts could reduce pipeline erosion. Tapes with three geometries (one twist, four twists, and four twists with holes) were installed in PVC pipes carrying sand concentrations of 1%, 3%, and 5%. Comparisons of experimental data with CFD-DPM predictions showed that tape geometry and flow conditions act as the main factors in reducing erosion and improving the pipe’s performance under abrasive conditions.

Based on the results of the experiments, simulations, and analyses conducted, several conclusions can be drawn:

1. Experimental results showed that twisted tape inserts greatly reduced paint erosion compared to plain pipes. Higher twist ratios and perforated tapes improved the distribution of abrasive particles, which reduced localized abrasion. The perforated four-twist tapes were the most effective, causing the least paint loss. Erosion rates increased with the sand concentration (from 1% to 5%), showing that particle concentration had a strong effect on erosion.
2. The mass loss assessment indicated that using twisted tape inserts reduced erosion and that the perforated four-twist tape offered the best performance. The one-twist tape reduced mass loss by 18%, the four-twist by 39%, and the perforated four-twist by 45% compared to the plain pipe control. Thickness loss was measured at 0.001 mm for the four-twist tape and 0.004 mm for the plain pipe. At the 1% sand concentration, thickness loss was reduced from 0.095% to 0.023%. At 5% concentration, the maximum loss was 0.21% in the plain pipe and only 0.095% with the four-twist tape.
3. SEM analysis confirmed that the perforated four-twist tape effectively reduced surface erosion, with fewer pits and shallower scratches. Tapes minimized deformation of the material by reducing adhesion, whereas perforations enhanced turbulent flow and reduced particle heat transfer.
4. Material hardness increased with the mixture’s concentration due to strain hardening. In the plain tube, the particles hit the pipe at full momentum and increased the hardness to 86. The one-twist tape decreased hardness by 2.9% (83.5), while the four-turn tape decreased it by 7.0% (80.9). The four-twist tape with perforations led to a further slight drop in hardness to 80.5. These results indicate that the twisted tapes help to maintain the hardness of the material while improving the flow properties.
5. The results of CFD-DPM simulations were in good agreement with the experimental data. Some disparities were observed due to the influence of parameters such as particle–particle interactions and sand phases, but the patterns of erosion reduction were similar. Incorporating twisted tapes, especially those with advanced geometries, reduced erosion rates in both the experimental and numerical analyses.
6. The current findings are based on laboratory-scale experiments carried out over a constrained range of flow conditions, particle sizes, and sand concentrations. Although they offer comparative trends, the paint-based mass loss and thickness measurements might not accurately reflect long-term industrial erosion behavior. While short exposure times and localized strain hardening may affect hardness changes, SEM observations provide qualitative insight into wear mechanisms. Furthermore, the CFD–DPM model ignores dense-phase effects and particle–particle interactions, which could account for small differences from experimental findings.

## 5. Future Work

Considering the findings of this study, we conclude that twisted tapes—specifically those with higher twist ratios and perforations—reduce pipeline erosion, especially for systems carrying abrasive materials such as slurries. In the above analysis, the four-twist tape with holes reduced the erosion rate more than the one-twist and four-twist tapes by maximizing swirling and turbulence. These openings enabled the fluid to pass but ensured its rotation, consistently distributing abrasive particles and minimizing their contact with the surface. In conclusion, the four-twist tape with holes emerged as the most protective against erosion. The current work should be expanded in the future to include multiphase flow modeling with CFD-DEM to observe interactions between particles and phases with greater solid loadings. The pressure drop and hydraulic efficiency should be experimentally and numerically validated to measure the trade-off between the reduction in erosion and energy losses caused by twisted tape inserts. Moreover, coupled CFD–experimental investigations can be employed to characterize flow uniformity, turbulence intensity, and particle paths, thus optimizing the inserts’ geometries to reduce erosion, as well as optimize hydraulic performance under industrial operating conditions.

This study has some limitations. First, some particle parameters in the models were fixed, while sand size varied. Second, instances of leakage occurred that could have affected the accuracy of the study. Third, the 3D-printed PLA inserts may not perfectly mimic industrial materials and geometries. Fourth, tests were conducted on relatively limited twisted tapes. Possible directions for future investigations may include determining the effects of the twist ratio, perforation size, materials used, and particle types on the erosion process. Research on multi-tape inserts, actual conditions, and longevity with reference to sustainability could contribute to innovative twist tape solutions required for industrial applications.

## Figures and Tables

**Table 1:** Composition of carbon steel (AISI 1040) [22].

Elements	% Composition
Carbon, C	0.37–0.44
Iron, Fe	98.6–99
Manganese, Mn	0.60–0.90
Phosphorous, P	≤0.040
Sulfur, S	≤0.050

**Table 2:** Simulation parameters [22].

Parameters	Setup
Material type (twisted tape)	Poly Lactic Acid (PLA)
Material type (test plate)	Carbon Steel (AISI 1040)
Concentration of sand	1%/3%/5% (wt./wt.)
Turbulence model	Reynolds Stress Model
Residuals	1 × 10^−5^
Pressure–velocity coupling scheme	SIMPLEC
No. of iterations	1000

**Table 3:** Summary of experimental cases [22].

Sample Type	Case No.	Concentration of Sand	Type of Twisted Tape Used
	1	1%	Plain Pipe
	2	1%	1-Twist
	3	1%	4-Twist
	4	1%	4-Twist–Holes
	5	3%	Plain Pipe
Painted	6	3%	1-Twist
	7	3%	4-Twist
	8	3%	4-Twist–Holes
	9	5%	Plain Pipe
	10	5%	1-Twist
	11	5%	4-Twist
	12	5%	4-Twist–Holes
	13	1%	Plain Pipe
	14	1%	1-Twist
	15	1%	4-Twist
	16	1%	4-Twist–Holes
	17	3%	Plain Pipe
Polished	18	3%	1-Twist
	19	3%	4-Twist
	20	3%	4-Twist–Holes
	21	5%	Plain Pipe
	22	5%	1-Twist
	23	5%	4-Twist
	24	5%	4-Twist–Holes

**Table 4:** Masses before and after the experiment [22].

	1%			3%			5%		
	I.W	F.W	Diff	I.W	F.W	Diff	I.W	F.W	Diff
Plain	41.385	41.201	0.184	43.033	42.752	0.281	42.698	42.32	0.378
1-Twist	43.888	43.74	0.148	42.571	42.367	0.204	39.098	38.835	0.263
4-Twist	43.392	43.28	0.112	43.468	43.306	0.162	43.761	43.534	0.227
4-Twist–Holes	43.275	43.17	0.105	39.512	39.362	0.150	40.105	39.9	0.205

**Table 5:** Thickness loss after the experiment [22].

Types of Twisted Tapes Used	1% Conc. (mm)	3% Conc. (mm)	5% Conc. (mm)
Plain	4.196	4.194	4.191
1-Twist	4.197	4.195	4.193
4-Twist	4.198	4.197	4.195
4-Twist–Holes	4.199	4.198	4.196

**Table 6:** The Vickers hardness values of all experimental samples [22].

Types of Twisted Tapes Used	1% Conc.	3% Conc.	5% Conc.
Plain	84.68	85.74	87.77
1-Twist	81.48	83.2	85.81
4-Twist	78.3	81.29	83.17
4–Twist–Holes	78.11	81.05	82.89

**Table 7:** The difference in erosion rates between experimental and CFD-DPM results for the plain pipe [22].

Plain Pipe	Exp Results	CFD-DPM Results	Diff	% Error
**1% Conc.**	0.184	0.248	0.064	0.25
**3% Conc.**	0.281	0.354	0.073	0.20
**5% Conc.**	0.378	0.438	0.060	0.13

**Table 8:** Differences in erosion rates between experimental and CFD-DPM results for 1-twist tape [22].

Twist Tape (1-Twist)	Exp Results	CFD-DPM Results	Diff	% Error
**1% Conc.**	0.148	0.213	0.065	0.30
**3% Conc.**	0.204	0.265	0.061	0.23
**5% Conc.**	0.263	0.320	0.057	0.17

**Table 9:** Difference in erosion rates between experimental and CFD-DPM results for 4-twist tape [22].

Twisted Tape (4-Twist)	Exp Results	CFD-DPM Results	Diff	% Error
**1% Conc.**	0.112	0.180	0.068	0.37
**3% Conc.**	0.162	0.216	0.054	0.25
**5% Conc.**	0.227	0.285	0.058	0.20

**Table 10:** Difference in erosion rates between experimental and CFD-DPM results for 4-twist tape with holes [22].

Twist Tape (4-Twist with Holes)	Exp Results	CFD-DPM Results	Diff	% Error
**1% Conc.**	0.105	0.169	0.064	0.37
**3% Conc.**	0.150	0.208	0.058	0.27
**5% Conc.**	0.205	0.262	0.057	0.21

**Table 11:** Comparison of experimental and CDF-DPM results [22].

	1%		3%		5%	
	Exp. Results	CFD-DPM Results	Exp. Results	CFD-DPM Results	Exp. Results	CFD-DPM Results
Plain	0.184	0.248	0.281	0.354	0.378	0.438
1-Twist	0.148	0.213	0.204	0.265	0.263	0.320
4-Twist	0.112	0.180	0.162	0.216	0.227	0.285
4-Twist–Holes	0.105	0.169	0.150	0.208	0.205	0.262

## Data Availability

The original contributions presented in this study are included in the article. Further inquiries can be directed to the corresponding author.
